# Biometric Evaluation of Baumann Skin Typing

**DOI:** 10.1111/srt.70338

**Published:** 2026-04-13

**Authors:** Shohreh Delavar, Aireza Firooz, Mansour Nassiri Kashani, Saman Ahmad Nasrollahi, Fatemeh Amiri, Taraneh Yazdanparast

**Affiliations:** ^1^ Center For Research and Training in Skin Diseases and Leprosy Tehran University of Medical Sciences Tehran Iran; ^2^ Dermatology Department, Faculty of Medicine, Tehran Medical Sciences Islamic Azad University Tehran Iran

**Keywords:** baumann skin typing, biometric assessment, skin typing

## Abstract

**Introduction:**

With the increasing demand for personalized skincare, accurate identification of individual skin types has become essential. The Baumann skin type (BST) classification system is widely used in clinical and commercial settings, yet lacks sufficient biometric validation. This study aims to evaluate the correlation between BST categories and objective skin biophysical parameters.

**Materials & methods:**

Seventy‐one healthy volunteers participated in the study. Skin parameters including stratum corneum hydration, transepidermal water loss (TEWL), melanin index, sebum levels, elasticity, and pH were measured using the MPA 580 system. Skin thickness and echodensity of the epidermis and dermis were assessed via high‐frequency ultrasound (HFU), while depth, area, and volume of the nasolabial folds were analyzed using VisioFace CSI software. Each participant completed the BST questionnaire, and correlations between BST scores and measured skin parameters were assessed using Pearson's correlation coefficient.

**Results:**

The oily vs. dry score was correlated with hydration (r = 0.08, *p* = 0.507), TEWL (r = ‐0.065, *p* = 0.591), and sebum (r = 0.299, *p* = 0.011). The sensitive vs.resistant score correlated with TEWL (r = 0.388, *p* = 0.001) and pH (r =  0.445, *p* < 0.001). The pigmented vs. non‐pigmented score correlated with melanin index (r =  0.280, *p* = 0.018). The wrinkle vs. tight score was correlated with skin firmness (R0; r = 0.495, *p*< 0.001), total skin elasticity (R2; r = ‐0.318, *p* = 0.007), dermal thickness (r = ‐0.242, *p* = 0.042), dermal density (r = ‐0.189, *p* = 0.115), epidermal thickness (r = ‐0.128, *p* = 0.288), nasolabial fold volume (r = 0.138, *p* = 0.250), surface (r = 0.150, *p* = 0.213), and depth (r = 0.048, *p* = 0.690).

**Conclusion:**

The oily vs. dry (O/D) score showed a poor but significant correlation with sebum, while hydration and TEWL were not significant. The pigmented vs. non‐pigmented (P/NP) score demonstrated a poor but significant correlation with the melanin index. The sensitive vs. resistant (S/R) score showed fair and significant correlations with TEWL and pH. The wrinkle‐tight (W/T) score showed fair and statistically significant correlations with elasticity parameters (R0 and R2), while correlations with nasolabial fold parameters, dermal density, and epidermal thickness were not significant; dermal thickness showed a poor but statistically significant correlation.

## 1 Introduction

Skin typing plays an essential role in modern dermatology and cosmetic science. Accurate identification of an individual's skin type informs not only treatment choices for dermatological conditions such as acne, eczema, and photodamage but also the selection of suitable skincare products and procedures [1, 2, 3, 4]. As public interest in personalized skincare continues to rise, there is increasing demand for reliable skin typing methods that go beyond general classifications [5].

Several classification systems have been developed to characterize different skin features, including the Fitzpatrick skin type, Lancer ethnicity scale, Roberts skin type, and the Japanese skin type classification [6]. Among these, the Baumann skin typing (BST) system—introduced by Dr. Leslie Baumann in 2006—is notable for its broader and more functional approach. The BST system uses the Baumann skin type questionnaire (BSTQ), a 64‐item self‐report instrument, to classify individuals across four dichotomous dimensions: oily (O) versus dry (D), sensitive (S) versus resistant (R), pigmented (P) versus nonpigmented (N), and wrinkled (W) versus tight (T). These combinations yield 16 unique skin types, offering more personalized and clinically actionable profiles compared to traditional models such as the Fitzpatrick scale, which focuses primarily on pigmentation and UV reactivity [7, 8].

Despite its popularity and practical utility in both clinical dermatology and the cosmetic industry, the BSTQ is based solely on subjective self‐assessment. This raises concerns about its objectivity and accuracy, especially given the increasing availability of noninvasive diagnostic tools capable of quantitatively assessing the very skin characteristics the BSTQ aims to capture.

Recent technological advancements in skin imaging and measurement have enabled precise evaluation of multiple skin biophysical parameters using devices such as the Sebumeter, Corneometer, Tewameter, pH meter, Mexameter, and Cutometer, as well as high‐resolution techniques like high‐frequency ultrasound (HFU) and VisioFace. These tools assess sebum levels, hydration, transepidermal water loss (TEWL), pH, melanin index, elasticity, and wrinkle morphology—metrics that align closely with the four BSTQ domains. Their proven reproducibility and validation in dermatological research position them as ideal instruments for evaluating the biometric correlates of BSTQ‐based classifications.

While the BSTQ offers a practical, cost‐effective, and accessible approach to skin typing, its validity has not yet been formally assessed using biometric methods. To address this critical gap, the present study aims to investigate the correlations between the four primary BSTQ parameters and corresponding biophysical skin measurements obtained through validated, noninvasive tools. To our knowledge, this is the first comprehensive attempt to objectively assess all four domains of the Baumann classification system using quantitative skin analysis

## 2 Material and method

### 2.1 Study Design and Population

This analytical single‐center clinical study was conducted at the Center for Research and Training in Skin Disease and Leprosy (Tehran University of Medical Sciences) between March 2021 and April 2024. Patients completed the BSTQ and were then referred for biometric characteristic evaluation using a multiple adapting system (MPA), Visio Face 1000 D and complete skin investigation (CSI), and the skin ultrasound imaging system (TPM, Germany). The study was conducted following the declaration of Helsinki and approved by the Ethics Committee of Tehran University of Medical Sciences (ethics code: IR.TUMS.MEDICINE.REC.1399.1170). Inclusion criteria were age over 18 years and informed patient consent to participate in the study. Exclusion criteria included individuals who had undergone any facial dermatological or cosmetic procedures (botulinum toxin, filler, etc) in the past six months, those using systemic steroid, topical steroid, or retinoid treatment within 6 months, and those who were pregnant or lactating. Demographic information including age and gender was collected. The Fitzpatrick skin type of each individual was recorded.

### 2.2 Assessment of Skin Profile

#### 2.2.1 Determination of Baumann Skin Type (BST)

The patients filled out the BSTQ, which has been described in detail elsewhere [9]. Scores for the four BST parameters (dry or oily, sensitive or resistant, pigmented or nonpigmented, and wrinkled or tight) were recorded for each patient. In group 1, higher scores indicate oilier skin; in group 2, higher scores indicate more sensitive skin; in group 3, higher scores indicate more pigmented skin; and in group 4, higher scores indicate wrinkled skin.

#### 2.2.2 Biometric Assessment

Patients were instructed not to apply any medicinal, cosmetic, or hygiene products to their skin from the night before the evaluation. On the day of the evaluation, participants rested for 20 min in an environment with controlled temperature (20°C–22°C) and humidity (30%–40%) before testing. Biometric assessment was performed using the MPA, a comprehensive tool consisting of a base unit and multiple probes.

For the oily versus dry dimension, three key parameters were measured: stratum corneum hydration using a Corneometer CM 825 probe, sebum secretion using a Sebumeter SM 815 device, and TEWL using a Tewameter TM 300 device. Sebum levels indicate oily skin, while lower hydration level and increased TEWL suggests drier skin.

The sensitive versus resistant dimension was evaluated based on skin surface pH (measured using a Skin‐pH‐Meter PH 905 device) and TEWL. Elevated pH and TEWL levels are considered indicators of impaired epidermal barrier function, and therefore, skin sensitivity.

For the pigmented versus nonpigmented classification, melanin content was quantified using a Mexameter (MX 18 device). This device emits light at specific wavelengths and measures melanin based on light absorption and reflection, providing an objective melanin index.

The wrinkled versus tight parameter was assessed using two complementary modalities. Skin elasticity was measured using a Cutometer 580 device focusing on the parameters *R*_0_ (representing skin firmness; higher values indicate less firm skin) and *R*_2_ (total elasticity; lower values suggest loss of elasticity). Measurements were performed with a 2‐mm aperture probe, applying a peak negative pressure of 500 mbar, a time under pressure of 2.0 s, and a time to ramp to peak pressure of approximately 0.3 s, in cycles of 2.0 s suction and 2.0 s relaxation, repeated five times. In parallel, epidermal and dermal thickness and density were assessed via high‐frequency ultrasound HFU imaging (TPM, Germany; DUB SkinScanner, 22MHz), which offers structural insights into age‐related skin changes. Additionally, wrinkle morphology—including depth, area, and volume—was quantified using VisioFace CSI software.

All measurements were taken at four standardized facial sites: the middle forehead, nose, chin, and cheeks. A single measurement was collected at each site, and the values were average across the four location to obtain a single value for analysis. Because there is a relationship between skin parameters and anatomical locations, using the mean of skin parameters measured on the middle forehead, chin, nose, and cheeks can enhance the sensitivity of the results applied in this study [10].

All devices (VisioFace 1000D, Tewameter, Cutometer, Mexameter, Sebumeter, and Skin‐pH‐Meter) were manufactured by Courage + Khazaka Electronic GmbH, Cologne, Germany.

## 3 Statistical Analysis

Data were analyzed using IBM SPSS Statistics 24 (SPSS Inc., Chicago, IL, USA). Mean and standard deviation (SD) were used for the description of quantitative data. The correlation between the score of each Baumann skin parameter and the mean of relevant skin parameters was determined using the Pearson correlation coefficient statistical method. The *p*‐values were obtained as part of the Pearson correlation test and were used to assess the statistical significance of the correlations (*p* < 0.05 was considered statistically significant).

The strength of correlation was based on the most widely used guideline in the medical field:

>0.8 very strong; ≥0.6 and ≤0.8 moderate; ≥0.3 and <0.6 fair; <0.3 poor.

## 4 Result

Seventy‐one patients were included in the study. All patients were women, of Iranian Asian descent, and had Fitzpatrick skin types 2,3, and 4.The mean age of the patients was 41.35 ± 11.01 years (range 18–62).

Based on the responses to the BSTQ, 28 participants (39.4%) reported having oily skin, 43 participants (60.6%) described their skin as dry. As for skin sensitivity, 44 participants (62%) considered their skin sensitive, while 27 participants (38.0%) described it as resistant. In terms of pigmentation, 34 participants (47.9%) reported having pigmented skin, while 37 participants (52.1%) noted that their skin was nonpigmented. Finally, when asked about skin texture, 27 participants (38.0%) reported having wrinkles, while 44 participants (62%) described their skin as tight.

The Pearson correlation between sebum levels and BSTQ oily/dry scores was statistically significant and poor in strength (*r* = 0.299, *p* = 0.011). Correlations between TEWL and stratum corneum hydration with BSTQ oily/dry scores were not statistically significant and showed poor relationships (*r* = –0.065, *p* = 0.591; *r* = 0.080, *p* = 0.507, respectively).

For the sensitive/resistant dimension, both TEWL and skin pH showed statistically significant and fair correlations with BSTQ scores (*r* = 0.388, *p* = 0.001; *r* = 0.445, *p* < 0.001, respectively).

Melanin concentration measured by the Mexameter had a statistically significant but poor correlation with the pigmented/nonpigmented BSTQ dimension (*r* = 0.280, *p* = 0.018).

Regarding the wrinkled/tight dimension, skin firmness (*R*_0_) showed a statistically significant fair correlation (*r* = 0.495, *p* < 0.001), while total skin elasticity (*R*_2_) demonstrated a significant but fair negative correlation (*r* = –0.318, *p* = 0.007).

Dermal thickness exhibited a poor but statistically significant negative correlation with BSTQ wrinkled/tight scores (*r* = –0.242, *p* = 0.042), whereas dermal density and epidermal thickness showed poor and nonsignificant correlations with BSTQ wrinkled/tight scores (*r* = –0.189, *p* = 0.115; *r* = –0.128, *p* = 0.288, respectively).

Nasolabial fold parameters (volume, surface area, and depth) showed poor and nonsignificant correlations with BSTQ wrinkle scores (*r* = 0.138, *p* = 0.250; *r* = 0.150, *p* = 0.213; *r* = 0.048, *p* = 0.690, respectively).

The results of the Pearson correlation analyses between biometric parameters—including sebum level, TEWL, pH, melanin concentration, skin elasticity, and skin thickness—and BSTQ scores are presented in Table 1.The mean values of each biometric measurement are summarized in Table 2.

**TABLE 1:** Correlation of BSTQ Scores with Biophysical Parameters for Different Skin Characteristics.

Skin characteristic	Biophysical parameter(Unit)	Measurement method	Correlation	*P*‐value
Oily/dry	Sebum(ug/cm2)	Sebumeter	0.299	0.011
	TEWL(g/m2/h)	Tewameter	−0.065	0.591
	SC hydration(arbitrary)	Corneometer	0.080	0.507
Sensitive/resistant	PH(arbitrary)	PH meter	0.445	0.000
	TEWL^b^	Tewameter	0.388	0.001
Pigmented/non‐ pigmented	Melanin level(arbitrary)	Mexameter	0.280	0.018
Wrinkled/tight	*R*_0_(arbitrary)	Cutometer	0.495	0.000
	*R*_2_(arbitrary)	Cutometer	−0.318	0.007
	Dermal thickness(um)	HFU^a^	−0.242	0.042
	Dermal density	HFU	−0.189	0.115
	Epidermal thickness(um)	HFU	−0.128	0.288
	Nasolabial fold volume(px3)	VisioFace	0.138	0.250
	Nasolabial fold surface(px2)	VisioFace	0.150	0.213
	Nasolabial fold depth(px)	VisioFace	0.048	0.690

^a^ HFU, high frequency ultrasound; ^b^TEWL, transepidermal water loss.

**TABLE 2:** Mean of skin biometric measurements

Biophysical parameters (Unit)	Mean	Std. Deviation
Nasolabial fold volume (px3)	42.35	39.38
Nasolabial fold surface (px2)	3.62	2.39
Nasolabial fold depth (px)	9.68	1.24
Transepidermal water loss (g/m2/h)	20.62	9.13
Sebum (ug/cm2)	89.91	54.77
SC hydration (arbitrary)	52.01	17.66
pH (arbitrary)	6.17	0.55
Melanin level (arbitrary)	198.77	50.68
Skin firmness (arbitrary)	0.2229	0.2148
Total skin elasticity (arbitrary)	0.5725	0.2540
Epidermal thickness (um)	108.68	11.97
Dermal density	30.70	8.11
Dermal thickness (um)	1387.28	202.72

## 5 Discussion

In this study, a statistically significant correlation coefficient (*p* < 0.05) is considered indicative of the questionnaire's validity in evaluating the corresponding skin parameter. Moreover, the magnitude of the correlation reflects the level of consistency and validity of the questionnaire in measuring that specific skin characteristic.

Oily skin is associated with increased sebum production. An increase in the D–O parameter score is expected to correspond with higher quantitative results from the sebumeter [11]. In our study, statistical analysis confirmed this correlation, with a correlation coefficient of +0.299. These findings suggest that the BSTQ shows a poor level of consistency with objective measures of oily skin.

Dry skin is characterized by a lack of water content in the stratum corneum and an increase in TEWL [12, 13]. According to the BSTQ, higher D–O parameter scores indicate less dry skin. Therefore, it is expected that higher D–O scores will correspond to lower TEWL values as measured by the Tewameter. Similarly, higher D–O scores are expected to correlate positively with increased stratum corneum hydration, as assessed by corneometer. Our study found correlations between the D–O score, TEWL, and stratum corneum hydration. However, these correlations were poor and statistically insignificant (*p* > 0.05). The analysis of these two parameters(TEWL and SC hydration)should have been restricted to the score range corresponding to the dry skin type in the BSTQ, as this approach could potentially enhance the sensitivity and accuracy of the evaluation.

Skin sensitivity is defined by the impairment of the skin barrier and vasculature hyperactivity [14, 15]. Elevated pH and increased TEWL are known indicators of a compromised skin barrier, often associated with sensitivity [16, 17]. A fair and statistically significant correlation was observed between skin pH and S‐R scores (*r* = 0.445), and TEWL also demonstrated a fair and statistically significant correlation (*r* = 0.388). These findings suggest that the BSTQ shows a fair level of consistency with objective features of skin sensitivity.

The skin pigmentation parameter (P‐N) in the BST system exhibits uneven skin tone due to overactive melanocytes [18]. Our study also demonstrated a poor statistical correlation between the melanin level and the P‐N parameter in the BSTQ, which was statistically significant. This indicates that the BSTQ shows a poor level of consistency with objective melanin levels.

It is well known that collagen and elastin are the main structural components of the skin that decrease with age [18, 19]. As a result, the BSTQ interprets higher scores in the W‐T (wrinkle–tightness) parameter as indicative of aging skin, which corresponds with reduced overall elasticity, as often seen in aging skin. In the Cutometer system, an increase in the *R*_0_ value reflects decreased skin firmness, as lower *R*_0_ values indicate tighter skin. Therefore, an expected relationship is that higher W‐T scores would be associated with higher *R*_0_ values, reflecting a decline in skin firmness. This relationship was confirmed in our study, where we observed a statistically significant fair correlation between the W‐T score and *R*_0_. Additionally, total skin elasticity is reflected by the *R*_2_ parameter in the Cutometer system, where lower *R*_2_ values indicate reduced overall elasticity. In line with expectations, we found a statistically significant fair correlation between W‐T scores and *R*_2_ values, suggesting that higher perceived aging in the BSTQ is associated with decreased total skin elasticity [20].

Aged skin, compared to young skin, is thinner and more fragile [21]. The HFU device enables the identification of changes in the extracellular matrix, including variations in dermal thickness and density, which are primarily associated with collagen [22]. Consequently, it is expected that an increase in the W‐T parameter score in the BSTQ would correlate with a decrease in the dermal thickness and density measurements obtained by HFU, which was confirmed in our study. A poor correlation was observed for both dermal thickness and density. However, for dermal thickness, the correlation was statistically significant, while for dermal density, it was not. On the other hand, the epidermal thickness becomes thinner with aging [23]. Therefore, it was anticipated that an increase in the W‐T score of BSTQ would lead to a decrease in epidermal thickness. This correlation was observed in our study which wasn't statistically significant. Our study shows that there is a poor correlation between the BSTQ wrinkle parameter and nasty fold parameters measured biometrically, which was not statistically significant. Taken together, the W‐T score of the BSTQ demonstrated fair and statistically significant correlations with two key objective indicators—*R*_2_ (total elasticity) and *R*_0_ (firmness). However, no significant correlations were found with other biometric aging markers.

Overall, our study showed that the BSTQ has statistically significant but poor to fair correlation in biometric evaluation, with four parameters determining skin type. Poor correlations were observed for oily skin, while measures for dry skin were not statistically significant. P/NP also showed poor correlation, whereas S/R and W/T demonstrated fair correlations, although some W/T measures were not statistically significant.

Our study supports a previous study in 2022 by Soo Ick Cho et al. They investigated the relationship between the results from the modified BSTQ and those from a digital photography analyzer and reported a positive correlation to some degree. However, in our study, we approached the original BSTQ and used a more precise device, the MPA system [24]. A study conducted in 2014 by L. S. Baumann et al. aimed to determinewhether the questions in sections O‐D of the BSTQ could successfully distinguish between individuals with high and low sebum secretion, as measured by a sebumeter [25]. The finding of their study indicates that the six oily skin‐directed questions of the BSTQ can classify individuals based on the level of sebum secretion. This result is consistent with our study on the validity of section O‐D of BSTQ in comparison to the sebumeter measurement.

## 6 Advantages, Limitations and Future Directions

Using validated skin type questionnaires based on biometric findings allows for the classification of skin types without the need for biometric devices, making it easier, more accessible, and cost‐effective for patients.

In addition to evaluating the current validity of the BSTQ, our findings suggest that improvements are needed across all domains of the questionnaire. The generally poor to fair correlations observed in this study indicate that a comprehensive revision may enhance the consistency of the BSTQ with objective skin characteristics. Strengthening the alignment between questionnaire responses and biometric measures will ultimately increase its clinical utility and accuracy in skin care decision‐making.

The current study encountered some limitations. First, only female participants were included, which allowed for better control of biological variability but may limit the generalizability of the findings to male populations. Second, all participants were recruited from a single center and had Fitzpatrick skin types II–IV, which may not represent broader populations. Future research should consider larger, more diverse populations, inclusion of male participants to validate our results.

## 7 Conclusion

This study investigated the relationship between the results from BSTQ and the objective and quantitative skin biophysical parameters. The oily vs. dry (O/D) score showed a poor but significant correlation with sebum, while hydration and TEWL were not significant. The pigmented va. non‐pigmented (P/NP) score demonstrated a poor but significant correlation with the melanin index. The sensitive vs. resistant (S/R) score showed fair and significant correlation with TEWL and pH. The wrinkle‐tight (W/T) score showed fair and statistically significant correlations with elasticity parameters (R0 and R2), while correlations with nasolabial fold parameters, dermal density, and epidermal thickness were not significant; dermal thickness showed a poor but statistically significant correlation.

## Data Availability

The data supporting the findings of this study are available from the corresponding author upon reasonable request.
